# Synergistic interactions of assorted ameliorating agents to enhance the potential of heavy metal phytoremediation

**DOI:** 10.1007/s44154-024-00153-1

**Published:** 2024-02-16

**Authors:** S. Sanjana, K. Jazeel, E. Janeeshma, Sarath G. Nair, A. M. Shackira

**Affiliations:** 1 Department of Botany, Sir Syed College, Kannur University, Kerala, 670142 India; 2 Department of Botany, MES KEVEEYAM College, Valanchery, Malappuram, Kerala India; 3 Department of Botany, Mar Athanasius College, Mahatma Gandhi University, Kottayam, Kerala India

**Keywords:** Chelators, Heavy metal, Metabolomics, Phytoremediation

## Abstract

Pollution by toxic heavy metals creates a significant impact on the biotic community of the ecosystem. Nowadays, a solution to this problem is an eco-friendly approach like phytoremediation, in which plants are used to ameliorate heavy metals. In addition, various amendments are used to enhance the potential of heavy metal phytoremediation. Symbiotic microorganisms such as phosphate-solubilizing bacteria (PSB), endophytes, mycorrhiza and plant growth-promoting rhizobacteria (PGPR) play a significant role in the improvement of heavy metal phytoremediation potential along with promoting the growth of plants that are grown in contaminated environments. Various chemical chelators (Indole 3-acetic acid, ethylene diamine tetra acetic acid, ethylene glycol tetra acetic acid, ethylenediamine-N, N-disuccinic acid and nitrilotri-acetic acid) and their combined action with other agents also contribute to heavy metal phytoremediation enhancement. With modern techniques, transgenic plants and microorganisms are developed to open up an alternative strategy for phytoremediation. Genomics, proteomics, transcriptomics and metabolomics are widely used novel approaches to develop competent phytoremediators. This review accounts for the synergistic interactions of the ameliorating agent’s role in enhancing heavy metal phytoremediation, intending to highlight the importance of these various approaches in reducing heavy metal pollution.

## Introduction

The rate of heavy metal pollution is increasing daily due to the uncontrolled discharge of sewage sludge and mining waste, excess use of chemical fertilizers and pesticides, intuitive industrial activities, etc. (Ekta and Modi 2018). At the same time, the macro and micro nutrients essential for plant growth include some heavy metals like cobalt (Co), copper (Cu), chromium (Cr), iron (Fe), nickel (Ni), manganese (Mn) and zinc (Zn). However, a higher concentration of these heavy metals imparts stress to the plants. There are some non-essential heavy metals like cadmium (Cd), mercury (Hg), arsenic (As), lead (Pb), etc. which are highly lethal to living organisms (Bhat et al. 2022). The bioavailability and toxicity of heavy metals mainly depend on the forms in which they are present in the environment (Shi et al. 2022). The physio-chemical processes such as ion exchange, precipitation, reverse osmosis, evaporation and chemical reduction are the remediation methods of heavy metal-polluted soil. However, the main problem is that these methods require external man-made resources and are too costly.

Plants play an important role in counteracting the harmful effects of these assorted pollutants to a greater extent in many ways. One promising aspect is the phytoremediation, which acts as a green surrogate to minimize the level of toxic metal ions with the employment of plants. Phytoremediation is an eco-friendly and low-cost method that can address the problems of metal pollution in a sustainable manner (Ekta and Modi 2018). In this emerging phytotechnology, various metal-tolerating plants are exploited for their ability to clean polluted soil or water.

In addition, some microorganisms, algae and other lower groups of plants, genetically engineered plants, etc., also help enhance the phytoremediation efficiency. The plant–microbe interaction is vital to proper plant growth, development and soil health (Rawool et al. 2020). In addition to this, some microorganisms like phosphate-solubilizing bacteria, endophytic microorganisms, mycorrhizas and plant growth-promoting rhizobacteria also exhibit the ability of heavy metal tolerance (Sruthi et al. 2016; Rawat et al. 2021; Sharma et al. 2021a, b). Nowadays, chemicals like Ethylene Diamine Tetra Acetic acid (EDTA), Ethylene Glycol Tetra Acetic acid (EGTA), Indole 3-Acetic Acid (IAA), Ethylenediamine-N, N′-disuccinic acid (EDDS) and Nitrilotri-acetic acid (NTA) are commonly used in order to enhance the efficiency of heavy metal phytoremediation. These chemicals interact with the rhizosphere and increase the mobility and metal uptake of plant roots. The synergistic interactions and cross-talk of soil microorganisms and various chemical chelators with the plant roots, which play a vital role in alleviating the stress and promoting the phytoremediation process, are detailed in the following sections.

## Phytoremediation-an overview

Phytoremediation refers to a diverse assemblage of work-based technologies that use naturally occurring or genetically engineered plants to clean contaminated environments (Flathman and Lanza 1998). The word ‘Phytoremediation’ is derived from the Greek prefix “phyto”, which means plant, and the Latin suffix “remedium”, which means clean or restore (Cunningham et al. 1997). Different plant-based technologies are grouped under phytoremediation– phytoextraction, phytostabilization, rhizofiltration, phytodegradation and phytovolatilization.

Phytoextraction is a method in which the metal absorbed by the plant is translocated to harvestable shoots and accumulates there. Phytostabilization differs from the rhizofiltration method in that it uses the plant to stabilize the contaminated soil rather than cleaning it. In contrast, in rhizofiltration, the plants are used to clean various aquatic environments. On the other hand, phytodegradation uses plants to uptake, metabolize and degrade the organic contaminants. Some plants extract certain volatile metals from soil and release them into the atmosphere through volatilization, known as phytovolatilization (Vasavi et al. 2010; Ekta and Modi 2018).

Plants chosen for phytoremediation should be fast-growing, high biomass yielding, high metal tolerating and hyperaccumulating, easily cultivatable and harvestable (Vasavi et al. 2010). There are many plants around us which have good phytoremediation potential and play a significant role in reducing the harmful effects of heavy metal pollution by performing various detoxification mechanisms like exclusion, excretion, accumulation, enzymatic and non-enzymatic anti-oxidant mechanisms, accumulation of osmoprotectants, chelation of metal by metallothioneins and phytochelatins, etc. (Sruthi et al. 2016).

## Amendments to enhance the phytoremediation efficiency

### Symbiotic microorganism

Microorganism-based bioremediation is a safe, inexpensive and eco-friendly option, and soil microbes promote/oppose or inhibit diverse biotic and abiotic processes, thereby sustaining the soil ecosystem (Dongre 2021). Plant microbiome interactions efficiently enhance the demolition of contaminants, incredibly toxic heavy metals, from the ecosystem by improving trace element uptake and translocation (Mocek-Plociniak et al. 2023). The microorganisms also assist the growth of plants grown in contaminated environments and mainly comprise phosphate-solubilizing bacteria (PSB), endophytic microorganisms, mycorrhizal associations and plant growth-promoting rhizobacteria (PGPR).

#### Phosphate-solubilizing bacteria (PSB)

During stressed conditions, using phosphate-solubilizing microorganisms is an eco-friendly approach to maintain agro-environmental sustainability. These microorganisms solubilize both inorganic and organic phosphorus through various mechanisms such as the production of organic acids, inorganic acids, hydrogen sulfide, siderophores, protons, excretion of extracellular enzymes; direct oxidation pathway and also through enzymatic actions (Yadav 2022). Additionally, these microbes not only solubilize phosphate but also promote plant growth and crop yield by producing plant growth-promoting hormones like auxins, gibberellins and cytokinins, antibiosis against pathogens, ACC deaminase (1-aminocyclopropane-1-carboxylate deaminase) which enhances plant growth under stress conditions, etc. thereby improving plant resistance to heavy metal toxicity (Rawat et al. 2021).

PSB abundance enhances the Cu-amelioration capacity of *Wedelia trilobata*, performing a higher rate of Cu absorption and translocation from contaminated soil (Lin et al. 2018). In high-Cd-mobilizing PSB, gluconic acid produced due to the peripheral peroxidation pathway is mainly responsible for high-Cd-dissolution. In low-Cd-mobilizing PSB, glycolic acid plays that role (Yang et al. 2018). For the remediation of Pb-contaminated sediments, PSB capsules coupled with phosphate materials are the most effective method (Zhang et al. 2020).

Along with a significant role in promoting various plant growth parameters, phosphate-solubilizing microorganisms also help to solve excess phosphorus contamination in phosphate mining wastelands by improving phytoremediation efficiency (Guo et al. 2021). The combined action of PSB and biochar-supported nano-hydroxyapatite results in the immobilization of Cd in contaminated river sediments and is a promising candidate for passivation material for sediment Cd (Zhao et al. 2022). Similarly, the application of PSB together with rice husk biochar in the soils that were heavily polluted by heavy metals like Pb/Cd helps in the reduction of soil acidification, enhancement of nutrients in the soil as well as total biomass of microbes within a short duration, that may also account for the reduced diffusion of toxic heavy metals (Lai et al. 2022). The application of PSB and plant growth-promoting rhizobacteria (PGPR) in *Vigna radiata* helps to reduce Cr toxicity to a great extent and also causes a tremendous increase in leaf number and area, length of root and shoot and chlorophyll synthesis (Mohanty and Mohapatra 2023).

#### Endophytic microorganism

The group of microorganisms colonized in the interior part of the plant, such as the root, stem or seeds and do not have any adverse effect on the host plant are referred to as endophytes (Rawool et al. 2020). Nowadays, endophytic microbes are explored mainly for their role in heavy metal stress mitigation in plant systems (Sharma et al. 2021a). Endophytic fungi provide enormous services to their host plants, including growth enhancement by nutrient acquisition, detoxification of heavy metals, secondary metabolite regulation and enhancement of abiotic/biotic stress tolerance (Khalid et al. 2021).

Endophytes isolated (*Enterobacter* species-LC1, LC4 & LC6; *Kocuria* species-LC2, LC3 & LC5 and *Kosakonia* species-LC7) from *Lantana camara* established within *Solanum nigrum* effectively improved plant growth besides increasing bioaccumulation and root to shoot transport of As when applied as consortium (Mukherjee et al. 2018). Endophytic bacterium *Sphingomonas* SaMR12 isolated from Cd/Zn hyperaccumulator *Sedum alfred*o improves the plant phytoextraction efficiency and heavy metal (Cd) tolerance. Hence these endophytes are considered an effective remediation candidate (Wang et al. 2020c). Some endophytic bacterial inoculation leads to the expression of heavy metal ATPase genes *(HMAs*) that encode heavy metal transport proteins. In rice seedlings, *HMA2*, *HMA3*, and *HMA4* play a leading role in Cd translocation (Ullah et al. 2022) (Table 1).

**Table 1:** Role of PSB in alleviating heavy metal phytoremediation potential by plants

PSB Strain	Heavy metal	Role	Reference
*Acinetobacter pittii* gp-1	Pb	Promote immobilization of Pb	Wan et al. 2020
*Acinetobacter pittii* and *Escherichia coli*	Cd	Motivated Cd accumulation in *Solanum nigrum* and promote plant growth	He et al. 2022c
*Leclercia adecarboxylata* L15	Pb	Release phosphate and formation of insoluble Pb-phosphate compounds resulted in Pb passivation in sediments	Zhang et al. 2020
*Pantoea* sp.PP4	Pb, Cd	Biosorption capability for Pb and bioprecipitation for Pb and Cd in *Lolium multiflorum*	Wei Xie et al. 2022
*Enterobacter ludwigii* GAK2	Cd	Reduced Cd content in rice plant	Adhikari et al. 2020
*Pseudomonas* sp. strain LA	Pb	Combination of strain LA, ryegrass and sonchus enhance the reduction of bioavailable Pb in soil from a phosphate mining waste land	Xiao et al. 2021
*Citrobacter farmeri* CFI-01	Pb	Immobilization of Pb was also ascribed to changes in the functional groups and the formation of lead phosphate sediments	Li et al. 2022c
*Acinetobacter pitti*	Cd	The combined action of fruit peel-based activator & PSB enhanced phytoextraction of Cd and growth of ryegrass	Zhao et al. 2023b
*Bacillus amyloliquefaciens* (CZ-B1)	Pb	The resistance of CZ-B1 to Pb is mainly achieved by cellular and secretions adsorption under low Pb stress	Zhang et al. 2023

#### Mycorrhizal association

Some fungi, namely mycorrhizas, are symbiotically associated with the roots of higher plants, and this positive interaction enhances water and nutrient uptake from the soil, thereby enhancing growth and yield. Mycorrhizal fungi form their extensive hyphal network in soil, and the extra-radical mycelia (ERM) serves as an artificial root system to increase nutrient uptake (Mahadevakumar and Sridhar 2020). Mycorrhizae comprises seven types of members: arbuscular, ecto, ectendo, arbutoid, monotropoid, ericoid and orchidaceous. Arbuscular and ectomycorrhizae are the most abundant and ubiquitous types (Parihar et al. 2020). Arbuscular mycorrhizal fungi (AMF) are associated with heavy metal absorption and tolerance in plants and hence act as stress alleviators by bioremediating soil polluted with heavy metal (Sruthi et al. 2016). The distinct feature of AMF is arbuscules, the nutrient exchange site between the host and fungi, which are also involved in metal uptake (Parihar et al. 2020).

The application of AMF, along with soil amendments, becomes the most effective strategy for heavy metal phytoremediation (Wang et al. 2022a). Similarly, inoculation of mycorrhiza and fly ash in *Acacia luederitzii* influences the dry matter accumulation by reducing the heavy metal (Cu, Ni, Pb, Mn and Zn) availability and metal uptake (Ultra and Manyiwa 2021). Various biochemical processes like metal detoxification, metal mobilization or immobilization, accumulation, transformation and translocation are facilitated by AMF, showing their beneficiary role in phytoremediation (Tiwari et al. 2020). The earthworm-AMF-plant symbiosis potentially plays an essential role in the phytoremediation of heavy metal-polluted soils (Wang et al. 2020a). AMF inoculation plays a vital role in the environment contaminated with As, Cd, Pb and Cr, in which AMF increases the accumulation rate of these metals in the roots of plants and increases the resistance of plants to the high toxicity of these metals, showing its enhanced phytoremediation efficiency (Boorboori and Zhang 2022). Moreover, the application of AMF and biochar in maize grown in the soils artificially contaminated with 5 mg Cd Kg^−1^ soil, is suitable for phytoremediation of Cd without much deleterious effects to the plant (Zhuo et al. 2020). At the same time it was also proved earlier that, in situations where the soil Cd concentration is 25 mg Kg^−1^, the ornamental plant Mirabilis jalapa can be successfully employed for the remediation of Cd contaminated soil (Wang and Liu 2014) (Table 2).

**Table 2:** Role of endophytic microorganism in alleviating heavy metal phytoremediation potential by plants

Endophytic microorganism	Type	Plant	Heavy metal	Role	Reference
*Mesorhizobium loti* HZ76 and *Agrobacterium radiobacter* HZ6	*Bacteria*	*Robinia pseudoacacia* L	Pb, Cu	Alleviate heavy metal stress	Fan et al. 2018
*Rhizobium leguminosarum* bv. *viciae*	*Bacteria*	*Pisum sativum* L	Cd	Alleviated Cd stress by producing the ACC deaminase enzyme	Belimov et al. 2019
*Sinorhizobium meliloti* CCNWSX0020	*Bacteria*	*Medicago sativa* L	Cu	Relieve the plant from Cu-induced toxicity, enhance phytostabilization of Cu	Duan et al. 2019
Trametes hirsute	*Fungus*	*Triticum aestivum* L	Pb	Displayed significant levels of Pb tolerance, increased the plant chlorophyll content and biomass	Malik et al. 2020
*Bacillus megaterium* sp. M002	*Bacteria*	*Sedum alfredii* Hance	Cd	Improved the root exudation along with an improvement in the root morphological traits which significantly contributed in the alleviation of Cd induced toxicity	Tang et al. 2019
*Stenotrophomonas maltophilia* R5-5	*Bacteria*	Rice	Cd	Reduced the Cd content in root and blade by 81.33 and77.78% respectively, down-regulated the expression of Cd transporters, OsNramp5 and OsHMA2 for alleviating the HM contamination	Zhou et al. 2020a, b
*Sphingomonas* SaMR12	*Bacteria*	*Brassica juncea* (L.) Czern	Cd	Enhanced root Cd accumulation, activate anti-oxidative response by decreasing concentration Of H_2_O_2_, MDA and proline, increase anti-oxidative enzymes and regulate GSH-AsA cycle	Wang et al. 2020a, b, c
*Pseudomonas putida* strain RE02	*Bacteria*	*Trifolium repens* L	Cd, Cr and Pb	Increased heavy metal detoxification ability	Liu et al. 2021
*Jeotgalicoccus huakuii*	*Bacteria*	*Cynodon dactylon* (L.) Pers	Hg	Showed complete Hg detoxification	Ustiatik et al. 2022
*Bacillus amyloliquefaciens*		*Eleusine indica* (L.) Gaertn			
*Buttiauxella, Pedobacter, Aeromonas eucrenophila* and *Ralstonia pickettii*	*Bacteria*	*Sedum plumbizincicola* X.H. Guo et S.B. Zhou ex L.H. Wu	Cd	Increased Cd content and enhanced the phytoremediation of Cd contaminated soil	Cheng et al. 2022
*Serratia* sp. AI001 and *Klebsiella* sp. AI002	*Bacteria*	*Oryza sativa* L	Cd	Induced Cd translocation, improved plant growth dynamics, relieved electrolyte leakage	Ullah et al. 2022
*Pseudomonas rhodesiae* GRC140	*Bacteria*	*Typhya latifoila* L	Cd	Increased the Cd content and accumulation in shoot	Rolon-Cardenas et al. 2022
*Bacillus* sp. AP10	*Bacteria*	*Arabidopsis thaliana* (L.) Heynh	Mn	Under Mn stress, they increased expression of key genes involved in cell wall loosening and phenylpropanoid pathway inorder to improve plant growth, promoted Mn uptake capacity of plant, alleviate Mn toxicity by enhancing ABA synthesis	Wu et al. 2023
*Aspergillus luchuensis* strain C7	*Fungi*	*Prosopis laevigata* (Humb. & Bonpl.ex Willd.) M.C.Johnst	Cu, Zn, Pb	Favoured the translocation of these metals from roots to leaves of the plant, exhibit increased Cu translocation ability, promote plant growth	Tovar-Sanchez et al. 2023

#### Plant growth promoting rhizobacteria (PGPR)

PGPR is a group of rhizobacteria that enhance plant growth and improve yield by producing various plant growth-promoting substances. PGPR is a biofertilizer and bioprotectant (Mahadevakumar and Sridhar 2020). PGPR has two modes of action- direct and indirect. Nitrogen fixation, phosphate solubilization and phytohormone production belong to the direct action. The indirect mechanism protects plants from plant pathogens by producing antimicrobial compounds (Glick 1995; Martinez-Martinez et al. 2023). Phosphate-solubilizing PGPR amends phytoextraction and phytostabilization efficiency of heavy metal treated plants. Using ACC deaminase activity, PGPR also enhances the growth of plants even in the presence of heavy metals (Kumar et al. 2023; Martinez-Martinez et al. 2023).

Multifunctional Plant growth promoting bacteria (PGPB)/PGPR showed Cr resistance and bio-inoculant properties with phytoremediation plants. By modifying root architecture and sequestering metals in the rhizosphere, PGPB enhances Cr uptake and lessens phytotoxicity. Growth regulators, mineral solubilizers, phytohormones and diverse secondary metabolites were produced by PGPB in order to speed up plant defence against metal poisoning (Dongre 2021). The phytoremediation efficiency of ryegrass on Cu-Cd co-contaminated soil can be improved by applying PGPR (Shi et al. 2022). The combined action of PGPR and salicylic acid (SA) in sunflowers helps to improve the heavy metal (Cd, Pb and Ni) phytoremediation efficiency and plant growth (Khan et al. 2018). PGPR adopt various defence mechanisms against heavy metal stress, such as compartmentalization, exclusion, complexity rendering, and the synthesis of metal-binding proteins (Sharma et al. 2021b) (Table 3).

**Table 3:** Role of mycorrhizae and PGPB in alleviating heavy metal phytoremediation potential by plants

Type		Plant	Role	Reference
**Mycorrhiza**	*Claroideoglomus etunicatum* BEG168	*Sorghum bicolor* (L.) Moench var. Yajin2	Potential for phytoremediation of Mo contaminated farmland and revegetation of Mo-mine disturbed areas, as well as biomass production on such sites	Shi et al. 2020
	*Funneliformis mosseae*	*Zea mays* L	Reduced the bioavailable Zn released from ZnO nanoparticles and decreased the concentrations and translocation of Zn to maize shoot	Wang et al. 2018a, b
			Act as bio-fertilizer in roots and modulate direct translocation of heavy metals like Cd, Cr, Ni and Pb	Singh et al. 2019
	*Funneliformis mosseae* (Fm), *Glomus versiforme* (Gv) and *Rhizophagus intraradices* (Ri)	*Zea mays* L	Decreasing Cd/Pb accumulation in maize and improve plant growth.	Zhuo et al. 2020
	*Funneliformis mosseae* and *Diversispora spurcum*	*Zea mays* L	Promoted the retention of heavy metal in roots and increased the uptake of Pb, Zn, Cd and As	Zhan et al. 2018
	*Acaulospora mellea* ZZ	*Sorghum bicolor* (L.) Moench	In combination with soil amendment (hydroxyapatite, manure & biochar) helps phytostabilization of Cd, Pb and Zn, promote plant growth	Wang et al. 2022a, b, c, d
	*Rhizophagus irregularis*	*Medicago sativa* L	Reduced shoot Cd concentration	Motaharpoor et al. 2019
	*Claroideoglomus claroideum* BEG210	*Helianthus annuus* L	Improve plant growth and phytostabilization efficiency in Ni contaminated soils	Ma et al. 2019a, b
	*Diversispora spurcum*	*Cynodon dactylon* (L.) Pers	Increased the uptake of Pb and Zn	Zhan et al. 2019
	*Rhizophagus intraradices*	*Oryza sativa* L	Decreased Cd uptake by altering the expression of Cd transporters	Chen et al. 2019a, b, c
	*Glomus aggregatum*	*Zea mays* L	AMF along with moderate amount of phosphorous may decrease Pb, Cd, and Zn uptake and increase plant growth	Nafady and Elgharably 2018
	*Glomus intraradices*	*Zea mays* L	Increased Hg uptake in roots	Debeljak et al. 2018
	*Glomus mosseae* and *Glomus intraradices*	*Robinia pseudoacacia* L	AMF significantly increased the efficiency of heavy metal phytoextraction	Zhao et al. 2023a, b
	*Funneliformis mosseae*	*Lavandula angustifolia* L	improved the phytoremediation potential of Pb and Ni	Rasouli et al. 2023
**PGPB**	*Brucella* sp. K12	*Hibiscus esculentus* L	Significant reduction in Cr(VI) (> 50% control) in soils and plants, thereby promote plant growth	Dongre 2021
	*Microbacterium* sp. SUCR140	*Pisum sativum* L	Reduce Cr(VI) toxicity by curtail its soil bioavailability and uptake in SUCR140-inoculated plants	
		*Zea mays* L	Cut Cr(VI) toxicity to plants & lower soil bioavailability, plant uptake via increased mycorrhizal colonization	
	*Paenibacillus lentimorbus* B-30488(r)	*Cicer arietinum* L	Reduce Cr(VI) uptake by plants & promote plant growth	
	*Ochrobactrum intermedium* C32413, *Ochrobactrum intermedium*	*Helianthus anuus* L	Reduce Cr(VI) uptake	
	*Ochrobactrum* CrT-1, *Bacillus cereus* S6	Mungbean	Reduce chromium toxicity to seedlings & carry reduction of Cr(VI) to Cr(III)	
	*Pseudomonas sp.* PsA4*, Bacillus sp.*Ba32	*Brassica juncea* (L.) Czern	Inspire plant growth & lessen Cr(VI) content	
	*Bacillus* species PSB10	*Cicer arietinum* L	Cut chromium uptake in roots, shoots and grains	
	*Agrobacterium tumefaciens*	*Zea mays* L	Increase plant biomass and Cr(VI) uptake	
	*Cellulosimicrobiumcellulans* KUCr3	Chilli	Reduce Cr uptake in plants	
	*Pseudomonas aeruginosa, Pseudomonas fluorescens, Ralstonia metallidurans*	Maize	Enhanced Cr uptake	
	*Enterobacter* sp. C1D	*Vigna radiate* GM4	Increased the plant tolerance towards Cr(VI), it also possessed the production of IAA at elevated levels and ACC deaminase activity during heavy metal toxicity	Subrahmanyam et al. 2018
	Azotobacter *chroococcum* CAZ3	*Zea mays* L	recovered the plant from heavy metal-induced oxidative damage by altering its root morphology, it also produced a melanin compound which revealed metal-chelating abilities	Rizvi and Khan 2018
	*Ensifer adhaerens* (strain OS3)	*Cicer arietinum* L	Displayed the retention of plant growth promotion traits on the plant exposure to higher concentrations of heavy metals and there by assuaged the Cd toxicity	Oves et al. 2017
	*Pseudomonas putida* KT2440	*Triticum aestivum* L	Resulted in potential increase of heavy-metal(Cd, Ag & Hg) phytoremediation from polluted soils, Enhanced expression of phytochelatin synthase genes	Yong et al. 2014
	*Planomicrobium chinense* (strain P1) and *Bacillus cereus* (strain P2)	*Helianthus annus* L	Enhance translocation & accumulation of heavy metals, production of IAA & GA helps the plant to tolerate stress	Khan et al. 2018
	*Pseudomonas aeruginosa*	*Lolium multiflorum* Lam	Improves the bioavailability of Cu & Cd thus enhancing the up-take of Cu & Cd by ryegrass and these metals transferred to shoot	Shi et al. 2022
	*Burkholderia* sp. D54	*Solanum lycopersicum* L	Decreased Cd concentration in aerial part & Cd translocation from roots to aerial parts	Guo et al. 2020
		*Lolium multiflorum* Lam	Increased Cd concentration in roots & aerial parts and Cd translocation from roots to aerial parts enhanced	
		*Glycine max* (L.) Merr	Decreased Cd concentration in roots & aerial parts	
	Enterobacter sp. FM-1	*Bidens pilosa* L	Increased the Cd extracted from the soil	Tang et al. 2023
	*Cupriavidus* sp. S-8–2	*Brassica napus* L	Reduced Sb-mediated oxidative stress and malondialdehyde contents by reducing Sb absorption, promote rape seedling growth under antimony(Sb) stress	Zheng et al. 2023

### Chemical chelators

The application of various chemicals is a promising approach to heavy metal phytoremediation when the heavy metal extraction by the plant is limited or poor (Hasan et al. 2019). When using chemical chelators for heavy metal remediation, the most crucial things considered are the plant type, application rate and chelate types (Baghaie and Polous 2019). Competition with other cations must also take into consideration when applying chemical chelators (Wang et al. 2022c). Several metal-chelating chemical agents have been supplemented in the soil to enhance the rate of metal detoxification (Table 4).

**Table 4:** Role of various chemical chelators in alleviating heavy metal phytoremediation potential by plants

Chemical	Plant	Result	Reference
EDTA	*Brassica juncea* (L.) Czern	Increased Pb uptake	Rathika et al. 2021
	*Sedum aizoon* L. and *Suaeda salsa* L	EDTA application promote these halophytes to absorb and enrich Pb & Cd in the contaminated soil	Wang et al. 2018a, b
	Bamboo	Increased the absorption of Pb^2+^ in all tissues with the higher concentration of Pb in root	Jiang et al. 2019
	*Datura stramonium* L	Enhanced phytoremediation of Cd	Shirkhani et al. 2018
	*Brassica juncea* L., *Brassica campestris* L. and *Brassica napus* L	Increase in root Cd concentration & Improve remediation of Cd-polluted soil	Dhaliwal et al. 2022
	*Sasa argenteostriata* (Regel) E.G. Camus	Phytoremediation of Pb, lower soil water-soluble Pb concentration when applied along with NTA	Yang et al. 2022a, b
	*Alcea rosea* (Linn.) Cavan. and *Hydrangea macrophylla* (Thumb.) Ser	Enhance the enrichment and transport capacity of Pb & Zn to promote phytoremediation	Duan et al. 2022
	*Ricinus communis* L	Enhanced the plant Pb & Cd absorption	Sarfraz et al. 2022
	*Dysphania ambrosioides* (L.) Mosyakin & Clemants	Enhances Cd phytoextraction	Jan et al. 2021
	*Lolium perenne* L	Enhanced the uptake of heavy metals(Zn, Cu, Ni, Cd, Pb) in sludge soil	Li et al. 2020a, b, c, d
	*Bryophyllum laetivirens* (Desc.) V.V.Byalt	Facilitates heavy metal(Cu, Pb, Zn, Cd, and Ni) uptake of root	Li et al. 2020a, b, c, d
	*Dahlia variabilis* Cav	Enhance phytoextraction of Cd & Pb	Alzahrani et al. 2020
	*Festuca arundinacea* Schreb	Enhanced Cd distribution into the dead leaves and significantly promoted Cd phytoextraction efficiency	Wang et al. 2019
	Poplar	Increased Cd accumulation	Dai et al. 2020
	*Vetiveria zizanioides* (L.) Nash	Complexed heavy metals and brought them into solution form and increased heavy metal (As, Cu, Mn, Ni, Pb, and Zn) uptake from the soil	Kereeditse et al. 2023
	Sunflower	Enhances the Cu absorption potential of sunflower and increases its tolerance to copper when applied along with IAA	Shah et al. 2023
IAA	*Sedum alfredii* Hance	Increase the uptake of heavy metals (Zn, Cd & Pb) in slightly contaminated soil	Chen et al. 2022
	*Typha latifolia* L	Increased Cd content &accumulation in root thereby increasing Cd immobilization in plant root	Rolon-Cardenas et al. 2022
	*Cyphomandra betacea* (Cav.) Sendtn	Decreased the Cd content	Li et al. 2020a, b, c, d
	*Cinnamomum camphora* (L.) Presl	Enhance Cd accumulation in leaves	Zhou et al. 2020a, b
	*Daucus carota* L	Enhanced Cd content in root	Faiz et al. 2022
	*Brassica juncea* L	Increased shoot uptake of Cd & uranium(U) and have maximum Cd & U removal efficiency	Chen et al. 2020a, b
	*Bryophyllum laetivirens* (Desc.) V.V.Byalt	Promote transport of heavy metals(Cu, Pb, Zn, Cd, and Ni) in plant	Li et al. 2020a, b, c, d
	*Sedum alfredii* Hance	Phytoremediation of Cd & Pb co-contaminated soil	Liang et al. 2021
	Sunflower	Increased the plant heavy metal(Pb & Cd) uptake significantly	Baghaie 2021
		Enhances the Cu absorption potential of sunflower and increases its tolerance to copper when applied along with EDTA	Shah et al. 2023
	*Silybum marianum* (L.) Gaertn	Reduced the Pb uptake	Bhardwaj et al. 2023
EGTA	*Solanum americanum* Mill	Enhance Cd removal efficiency	Sharma et al. 2022a, b
	Poplar	Increased Cd accumulation	Dai et al. 2020
	*Mirabilis jalapa* L	Enhancing Cd translocation efficiency	Wang & Liu 2014
	*Cicer arietinum* L	Decrease Cd accumulation	Sakouhi et al. 2016
	*Mirabilis jalapa* L	Improve the Cd capacity in shoots	Wei et al. 2018
	*Helianthus annuus* L. and *Brassica napus* L	Pb and Zn remediation	Baghaie and Polous 2019
	*Festuca arundinacea* Schreb	Enhanced Cd distribution into the dead leaves and promoted Cd phytoextraction efficiency	Wang et al. 2019
EDDS	*Solanum americanum* Mill	Enhance Cd removal efficiency	Sharma et al. 2022a, b
	*Medicago sativa* L	Enhance Zn phytoremediation	Wang et al. 2022a, b, c, d
	*Zebrina pendula* Schnizl	Enhance Cd & U phytoextraction	Chen et al. 2019a, b, c
	*Helianthus annuus* L	Enhance the phytoextraction of Pb &Cd	Moslehi et al. 2019
	*Astragalus sinicus* L	Enhanced phytoextraction of Co	Chen et al. 2019a, b, c
	*Helianthus annuus* L	Increased Cd accumulation	Xu et al. 2021
	*Medicago sativa* L	Enhancing Zn phytoextraction	Wang et al. 2021
	*Amaranthus hybridus* Linn	Raised the Cd concentration in plant shoot &root	Li et al. 2018
	*Lolium perenne* L	Phytoextraction of Cu and Zn ions	Borker et al. 2020
	*Lobularia maritima* (L.) Desv	Enhance phytoextraction of Co	Chen et al. 2020a, b
	*Astragalus sinicus* L	Enhance phytoextraction of Co- contaminated soil	Chen et al. 2019a, b, c
NTA	*Medicago sativa* L	Enhance Zn phytoremediation	Wang et al. 2022a, b, c, d
	*Eremochloa ophiuroides* (Munro) Hack	Decreased root Pb absorption & accumulation, enhance Pb translocation efficiency	Pu et al. 2022
	*Athyrium wardii* (Hook.)	Enhancing Pb remediation efficiency by increasing Pb availability in soil	Yu et al. 2020; Zhang et al. 2021
	*Sasa argenteostriata* (Regel) E.G. Camus	Enhancing Pb phytoremediation Efficiency	Yang et al. 2022a, b
	*Sorghum sudanense* (Piper) Stapf	Improved phytoextraction of Ni	Jiao et al. 2022
	*Panicum virgatum* L	Phytoextraction of Pb	Hart et al. 2022
	*Solanum americanum* Mill	Enhance Cd removal efficiency	Sharma et al. 2022a, b
	Canola	Ni phytoremediation	Baghaie and Daliri 2020
	*Lepidium sativum* L	Promoted Hg phytoextraction	Smolinska 2020
	*Zea* *mays* L	convert insoluble fractions of Cd into soluble forms and increase the removal efficiency of Cd in the phytoremediation method	Mehrab et al. 2023

#### Indole 3-Acetic Acid (IAA)

IAA is a plant growth regulator and is the natural form of auxin. It plays a significant role in improving plant growth and heavy metal phytoremediation potential. IAA induces the activation of ATPases in the plasma membrane, thereby producing changes in the transport of ions through the membrane, which are related to heavy metal accumulation (Ji et al. 2015).

For instance, exogenous application of IAA results in the rise of Cd immobilization in *Typha latifolia* root. The effect may be due to IAA-induced increased synthesis of cell wall components upon which Cd fixation occurs (Rolon-Cardenas et al. 2022). Upon Cd stress, exogenous IAA application enhanced peroxidase and superoxide dismutase activities in the leaves of *Cyphomandra betacea* seedlings and decreased soluble protein content (Li et al. 2020d). During Cd stress in *Cinnamomum camphora*, the external IAA application enhanced the photosynthetic rate by the increased biosynthesis of total chlorophyll and carotenoid content, reduced the level of proline, soluble sugar, MDA (malondialdehyde) content and was found more efficient for Cd phytoremediation (Zhou et al. 2020a). The ameliorative role of IAA and silver nanoparticles against Cd stress in carrots was shown by suppressing ROS (reactive oxygen species) overproduction, increased activities of antioxidant enzymes, and phenol synthesizing and oxidizing enzymes (Faiz et al. 2022). The combined action of IAA and oxalic acid in *Sedum alfredii* (Cd/Zn hyperaccumulator and Pb-accumulating plant) effectively enhances the phytoremediation potential of Cd and Pb co-contaminated soil (Liang et al. 2021).

#### Ethylene Diamine Tetra Acetic acid (EDTA)

EDTA is the most commonly used and potential organic ligand that immobilizes heavy metals, enhances the uptake of metals through roots in the form of soluble metal-EDTA complexes, and supports metal xylem loading (Hasan et al. 2019). EDTA shows a complex relationship with pH, and its metal detoxification efficiency is related to soil types (Subasic et al. 2022).

The application of EDTA, along with hormones like IAA and kinetin, reduced the adverse effects of Cd by increasing the total protein content and peroxidase activity (Shirkhani et al. 2018). EDTA enhances Pb’s availability, absorption and translocation in bamboo plants growing in Pb-contaminated soil (Jiang et al. 2019). The most advantageous approach for the remediation of Pb-contaminated soil is using EDTA and biochar because their combined action enhances the phytoextraction rate of Pb and promotes plant growth (Rathika et al. 2021). The environmental risk associated with excess EDTA application can be lowered by the co-action of EDTA with degradable chelating agents like nitrilotri-acetic acid (NTA), and their combined action also enhanced Pb remediation efficiency in the dwarf bamboo plants (Yang et al. 2022b).

#### Ethylene Glycol Tetra Acetic Acid (EGTA)

EGTA plays a positive role in plant heavy metal uptake and is widely used as a chelating agent (Hasan et al. 2019). EGTA plays a crucial role in Cd accumulation and can be enhanced by applying EDTA (Dai et al. 2020). EGTA show better performance than EDTA in Cd phytoextraction of the ornamental plant *Mirabilis jalapa* (Wang and Liu 2014; Wei et al. 2018). The application of exogenous EGTA and Ca in chickpea seeds alleviated Cd-induced growth damage and decreased lipid peroxidation and protein carbonylation in both shoots and roots (Sakouhi et al. 2016). The mechanisms induced by Ca and EGTA to protect the cell from Cd-induced oxidative injury include the triggered thiol-protecting process through activation of the Trx system and restoring the control level of antioxidative enzyme activities (Sakouhi et al. 2018). EGTA enhanced Cd accumulations in the dead leaves of tall fescue plants, which could be associated with the increase of the water-soluble inorganic Cd and Cd organic acid complexes in the shoots (Wang et al. 2019).

Upon supplementation of biodegradable chelates such as EGTA, EDDS, NTA and citric acid (CA) in *Solanum nigrum*, the EGTA application shows improved Cd phytoextraction efficiency compared to others, with an increased tolerance index value, transfer coefficient of root and translocation factor (Sharma et al. 2022b). For the removal of Cr(III) in a highly saline organic wastewater environment, EGTA-modified magnetic microspheres were used (Wang et al. 2020b).

#### Ethylenediamine-N, N′-succinic acid (EDDS)

EDDS is a biodegradable solid chelating agent. EDDS is produced by biological methods such as fermentation (in *Amycolatopsis japonicum* MG417-CF17) through the most economical, eco-friendly enzymatic methods in which ethylene diamine and fumaric acid is used as substrate (Wang et al. 2022d). When comparing the efficiencies of EDDS and NTA in enhancing the Zn phytoremediation by alfalfa, EDDS seems more efficient due to the higher Zn concentration in soil pore water induced by EDDS. EDDS can remediate uranium (U) and Cd in *Zebrina pendula*. However, it is equally efficient in Cd phytoextraction because of its more significant effect on shoot Cd accumulation. The ability of EDDS to activate Cd in soil was better than that of citric acid and oxalic acid treatments (Chen et al. 2019a).

The best amendment combinations for Pb phytoextraction are EDDS and vermicompost (Moslehi et al. 2019). When applied with 5-aminolevulinic acid (ALA), EDDS promoted Cd absorption and biomass accumulation in sunflowers growing on Cd-contaminated soil (Xu et al. 2021). The highest EDDS application leads to lower biomass production in alfalfa. Hence in order to minimize phytotoxicity and improve Zn phytoextraction efficiency in alfalfa, the EDDS dosage should be adjusted for each soil, depending on its characteristics and metal content (Wang et al. 2021). The application of plant growth regulators (diethyl aminopurine and 6-benzylaminopurine) along with EDDS mitigates the negative impact of EDDS on plant growth, resulting in enhanced Cd phytoaccumulation and translocation (Li et al. 2018).

#### Nitrilotriacetic acid (NTA)

NTA is an environmentally friendly, biodegradable chelating agent that strengthens phytoremediation (Pu et al. 2022). NTA is a derivative of EDTA. The biodegradable nature and reduced toxicity of NTA towards microorganisms and plants make it more advantageous in phytoextraction techniques (Hart et al. 2022). The lag phase for the degradation of NTA varied from 0–7 days (Wang et al. 2022c).

NTA play a vital role in mineral absorption and transportation in centipede grass, showing increased root Mg, K and Ca and shoot Fe, Cu and Mg concentrations (Pu et al. 2022). The chelating capability of NTA makes NTA-modified *Dendrocalamus strictus* charcoal powder a sound absorbent for the removal of Cu(II) ions from an aqueous solution (Saini et al. 2020). When NTA is applied along with EDTA, Pb remediation efficiency in dwarf bamboo gets boosted significantly (Yang et al. 2022b). The combined action of NTA and Triton-X-100, an alkyl polyglucoside (APG), increased the Pb concentration to more than double that in the foliage of switchgrass (Hart et al. 2022). The NTA application in *Athyrium wardii* modifies plant rhizosphere by lowering pH, increasing dissolved organic carbon, exudation and soil enzyme activities, These alterations contributed to the increased Pb accumulation (Zhang et al. 2021).

## Synergistic impact of chemicals and microbes in heavy metal stress tolerance

The independent application of chemical chelators and microbes for heavy metal tolerance is beneficial, but further improvement is possible through the synergic treatment of both chemical chelators and microbes. The synergistic application of chemical chelators and microbes aided in improving the bioavailability of some toxic metals as well as the microbial population of soil. The simultaneous application of AM fungus and EDTA improved the heavy metal tolerance of corn (*Zea mays*) and sunflower (*Helianthus annuus*), and Pb extraction was maximum in EDTA-applied soil (Usman and Mohamed 2009). So, synthetic chelators should increase the bioavailability of selective heavy metals, and the following application of microbes enhances the remediation potential. *Cronobacter sakazakii-* EDTA complex increased the phytoremediation potential of *Zea mays* L. to remediate Pb-contaminated soils (Menhas et al. 2021). *C. sakazaii*-EDTA (5 mM EDTA kg^−1^) complex aided the plant in tolerating metal toxicity by improving biomass production, synthesizing photosynthetic pigments, maintaining the water status, and accumulating proline. Moreover, maize plants showed differential tolerance levels towards different soil types, and spiked and aged soil showed different responses under the application of chelators (Menhas et al. 2021). This improvement in the tolerance level depends on the changes in the microbial population due to the soil washing with chelators. Members of *Nocardioidaceae* were prominent in the soil washed with 10 mmol kg^−1^ EDTA. However, the dominant microbial population was shifted to chemolithoautotrophic bacteria, such as *Nitrososphaeraceae*, in the soil washed with 60 mmol kg^−1^ EDTA (Wei et al. 2020). Thus, knowledge of the nature of the contaminated metal and the microbial population is essential for the better performance of chelators in phytoremediation.

## Transgenic approaches

The use of advanced technologies put forward various transgenic approaches to improve the efficiency of heavy metal phytoremediation. The crucial aspects taken into consideration while constructing genetically engineered organisms (GEOs) for the removal of pollutants include modification of enzymes, regulation/control of biological pathways, developments in affinity sensors, post-release monitoring of GEOs, application of molecular tools, risk assessments, pathogenesis, adverse environmental and health effects and biosafety issues (Iravani and Varma 2022). However, in the recent past, several genetically modified organisms have been engineered which have increased potential for metal detoxification without compromising the growth process (Table 5).

**Table 5:** List of transgenic organisms engineered so as to enhance the heavy metal phytoremediation potential

Transgenic approaches		Heavy metal	Gene	Reference
**Transgenic plants**	*Sedum plumbizincicoloa* X.H. Guo et S.B. Zhou ex L.H. Wu	Cd	**SpHMA1** (heavy metal ATPase 1 of *S plumbizincicoloa*)	Zhao et al. 2018
	Pusa-362 (*desi* Chickpea cultivar)	Pb & Cu	**CarMT1**(metallothionein type1)	Kumar et al. 2022
	Arabidopsis	Cd	**SlJMJ524**(from tomato)	Li et al. 2022a, b, c
	Tobacco	Cd	**Fld** (flavodoxin) & **BADH** (betaine aldehyde dehydrogenase)	Shahbazi et al. 2022
		Cd, Cu and Zn	**TdSHN1** (ethylene-responsive transcription factor of durum wheat)	Djemal and Khoudi 2022
		U	**cytc3** (cytochrome c3 from *Desulfovibrio vulgaris*)	Beliaev et al. 2021
		Zn	**SbMT-2**(cloned from *Salicornia brachiata*)	Patel et al. 2021
	Poplar	Cd	**PyWRKYJ5** (isolated & cloned from *Populus yunnanensis*)	Wu et al. 2022
	Wheat	Cd	**TaSWEET14**	Liu et al. 2022b
		Cd	**AetSRG1**(Fe(II)/2-oxoglutarate dependent dioxygenase)	Wei et al. 2022
	Barley	Cd	**HvNAT2**(Nucleobase-ascorbic acid transporters(NAT))	Wang et al. 2022b
	*Arabidopsis thaliana* (L.) Heynh	Cd	**ApHIPP3**(from *Arabis paniculata*)	Liu et al. 2022a
		Cr(VI), Cd, As(III)&As(V)	**MT1**(chickpea metallothionein1)	Dubey et al. 2021
			**CaGrx**(chickpea glutaredoxin)	Kumar et al. 2020
	*Populus alba* and *Arabidopsis thaliana* (L.) Heynh	Zn	**ScZRC1** (*Saccharomyces cerevisiae* ZRC1)	DalCorso et al. 2021
	Tobacco, Arabidopsis, Tomato and Rice	Hg	**merA** and **merB**	Li et al. 2020c
	Tobacco	Cd, Cu	***LmTrxh2*** (thioredoxin protein-encoding gene)	Ben Saad et al. 2023
**Transgenic microorganisms**	*Rhizobium leguminosarum* 3841-PsMT1	Cd	**PsMT1**(metallothionein from *Pisum sativum*)	Tsyganov et al. 2020
	*Rhizobium leguminosarum* 3841-PsMT2	Cd	**PsMT2**(metallothionein from *Pisum sativum*)	
	*Escherichia coli* MT3	Cd	**MT3**(human metallothionein)	Uckun et al. 2021
	*E. coli* MT2	Cd	**MT2**(human metallothionein)	
	*E.coli*	Cd, Cu & Zn	**ShMT**(metallothionein from *Sinopotamon henanense*)	Li et al. 2021
	*E.coli* (SynEc2)	Cd & Pb	**SynHMB** (synthetic heavy metal capturing gene containing metallothionein sequence) & T6SS (synthetic typeVI secretory system cluster of *Pseudomonas putida*)	Zhu et al. 2020
	*E.coli* BL21(DE3)	Pb(II)	**bmtA**(metallothionein from *Pseudomonas aeruginosa* N6P6)	Kumari and Das 2019
	*E.coli*	Cu, Cd & Zn	**MT**(metallothionein from freshwater crab)	Ma et al. 2019a
	*Rhodopseudomonas palustris*	Cd, Zn & Cu	**MT**(metallothionein from crab *Sinopotamon henanense*)	Jia et al. 2022
	*Saccharomyces cerevisiae*	As	**WaarsM**(encoding arsenic methyltransferase from *Westerdykella aurantiaca*)	Verma et al. 2019
	*Chlamydomonas reinhardtii*	As	**acr3**(from *Pteris vittata*)	Ramirez-Rodriguez et al. 2019
	Yeast	Cd, Cu	***LmTrxh2*** (thioredoxin protein-encoding gene)	Ben Saad et al. 2023

### Transgenic plants

Plants can be engineered to improve their ability to remediate metal pollution through the transfer and insertion of desirable genes from a foreign source into a plant of interest and produce transgenic plants with overexpression of the desirable genes like genes involved in metal uptake, translocation, sequestration, etc. (Placido and Lee 2022). The advantages of genetic engineering are the requirement of a short period and the ability to transfer desirable genes from hyperaccumulators to sexually incompatible plant species, and these are impossible in traditional breeding methods. While designing transgenic plants, selecting desirable genes and host plants are the main factors considered (Yan et al. 2020).

The merA and merB expressing transgenic plants (Arabidopsis, tobacco, tomato and rice) grown in Hg-contaminated soil can produce safe food like vegetables, fruits and grains for human and animal consumption (Li et al. 2020c). Regulation of the thiol-dependent mechanism helps to reduce the heavy metal toxicity in *Arabidopsis thaliana* and is achieved through the overexpression of the MT1 gene (Dubey et al. 2021). SlJMJ524 gene overexpression in Arabidopsis plants controls metal transporter-related gene expression as well as increased flavonoid content in plants, thereby exhibiting Cd tolerance during seedling and maturation stages (Li et al. 2022a).

Similarly, the glutathione derived phytochelatins (PC) molecules are usually synthesized when plants encounter heavy metal stress and its synthesis is mediated by the enzyme phytochelatin synthase (PCS). Their mode of action is in such a way that it binds to free metal ions and sequester it to the vacuoles (Sruthi et al. 2016; Yan et al. 2020; Zhu et al. 2021; Jin et al. 2022). The overexpression of PCS gene has a greater contribution to Cd tolerance in plants by regulating PC synthesis (Zhu et al. 2021; Jin et al. 2022). For example, the overexpression of Boehmerianivea derived PCS gene, BnPCS1 in Arabidopsis showed improved tolerance, accumulation and translocation of Cd along with the reduced cellular damages in these transgenic lines (Zhu et al. 2021). Similarly, the overexpression of maize ZmPCS1 gene in Arabidopsis enhanced Cd tolerance where as its ectopic expression in Arabidopsis mutant lines (atpcs1) helps to overcome the Cd hypersensitivity of atpcs1. Also its transient expression in tobacco reduced Cd toxicity (Jin et al. 2022).

Most of the transgenic plants show high proline content, antioxidant enzyme activities with lower hydrogen peroxide, MDA and decreased electrolyte leakage during heavy metal stress (Kumar et al. 2020; Djemal and Khoudi 2022; Kumar 2022 ; Shahbazi et al. 2022). In addition to the Cd tolerance in transgenic wheat, TaSWEET14 overexpression alters ion transporter gene expression, and TaSWEET14 expression is positively regulated by TaMYB41 at its transcriptional level. Likewise, AetSRG1 overexpression prevents degradation of phenylalanine ammonia-lyase (PAL) and programmed cell death in *Aegilops tauschii* (Liu et al. 2022a, b; Wei et al. 2022).

### Transgenic microorganism

Genetically engineered microbes (GEM) are constructed using recombinant DNA technology. A desirable gene from an organism of the same or different species is inserted into a microbial genome or plasmid (Verma et al. 2020). The main advantages of genetically engineered microorganisms for their use in heavy metal bioremediation are the cost-effectiveness, ecofriendliness, simplicity and upscalability. The genetically engineered bacteria can improve metal-chelating proteins, metal stress tolerance, bioaccumulation of heavy metal and overexpression of peptides thereby executing bioreduction and recovery of heavy metal ions (Iravani and Varma 2022). Genetic modification of channel proteins (belongs to heavy metal uptake and transport system) and metal binding entities (belongs to heavy metal storage system) like metallothionein (MT), phytochelatins (PC) and polyphosphates (PolyP) enhances heavy metal phytoremediation efficiency (Verma et al. 2020).

Genetically engineered bacteria expressing MT have been increasingly used to treat heavy metals. MT is a low molecular weight cysteine-rich proteins that enable them to readily bind and sequester metal ions (Tsyganov et al. 2020; Li et al. 2021; Uckun et al. 2021). The use of transgenic rhizobia in association with legumes to enhance phytoremediation efficiency is collectively called symbiotic engineering (Jach et al. 2022). The Cd removal rate of transgenic *E. coli* (MT3 and MT2) is affected by temperature, pH and contact time (Uckun et al. 2021). In the genetically engineered *E. coli*, the ShMT gene is modified by site-directed mutagenesis and recombinant proteins (ShMT1, ShMT2 and ShMT3 having one, two and three-point mutation respectively) were further enhanced using SUMO fusion expression system to yield SUMO-ShMT1, SUMO-ShMT2 and SUMO-ShMT3 having enhanced heavy metal binding capacities (Li et al. 2021). The coassembly of genetically engineered *E. coli* (SynEc2) and magnetic nanoparticles modified by polyethyleneimine and diethylene triamine pentaacetic acid captures heavy metals with high removal efficiency (Zhu et al. 2020). Heavy metal biosorption is facilitated by functional groups on the cell membrane of recombinant cells (Jia et al. 2022).

### Omics tools

Omics tools are novel approaches to develop competent phytoremediators and the technique may include genomics, proteomics, transcriptomics and metabolomics. Genomics DNA sequencing and analysis are carried out, whereas in proteomics, target protein identification, quantification, and expression analysis takeranscriptomics involves RNA sequencing, expression and regulation profiling. Metabolomics is an implicit tool for profiling metabolites, hormones and signalling molecules (Agarwal and Rani 2022; Anjitha et al. 2023).

Proteomics studies help to understand protein modification during Cd stress (Li et al. 2022b); Cd regulated transport proteins like ABC transporters, ion transport proteins, aquaporins, proton pumps and organic transport proteins (Zhu et al. 2022); protein abundance during Pb exposure such as increased abundance of hemicellulose and pectin related cell wall proteins for sequestration Pb thereby reducing its toxicity (Shen et al. 2021); effect of exogenous nitrogen on protein expression patterns under Cd stress (Zhang et al. 2022), etc. Gene structure, evolution and phylogenetics, chromosomal localization, gene doubling, cis-elements and expression profiles of genes during heavy metal stress were determined using genomics and bioinformatics (Gao et al. 2022; He et al. 2022a; Xie et al. 2022).

**Fig. 1:**
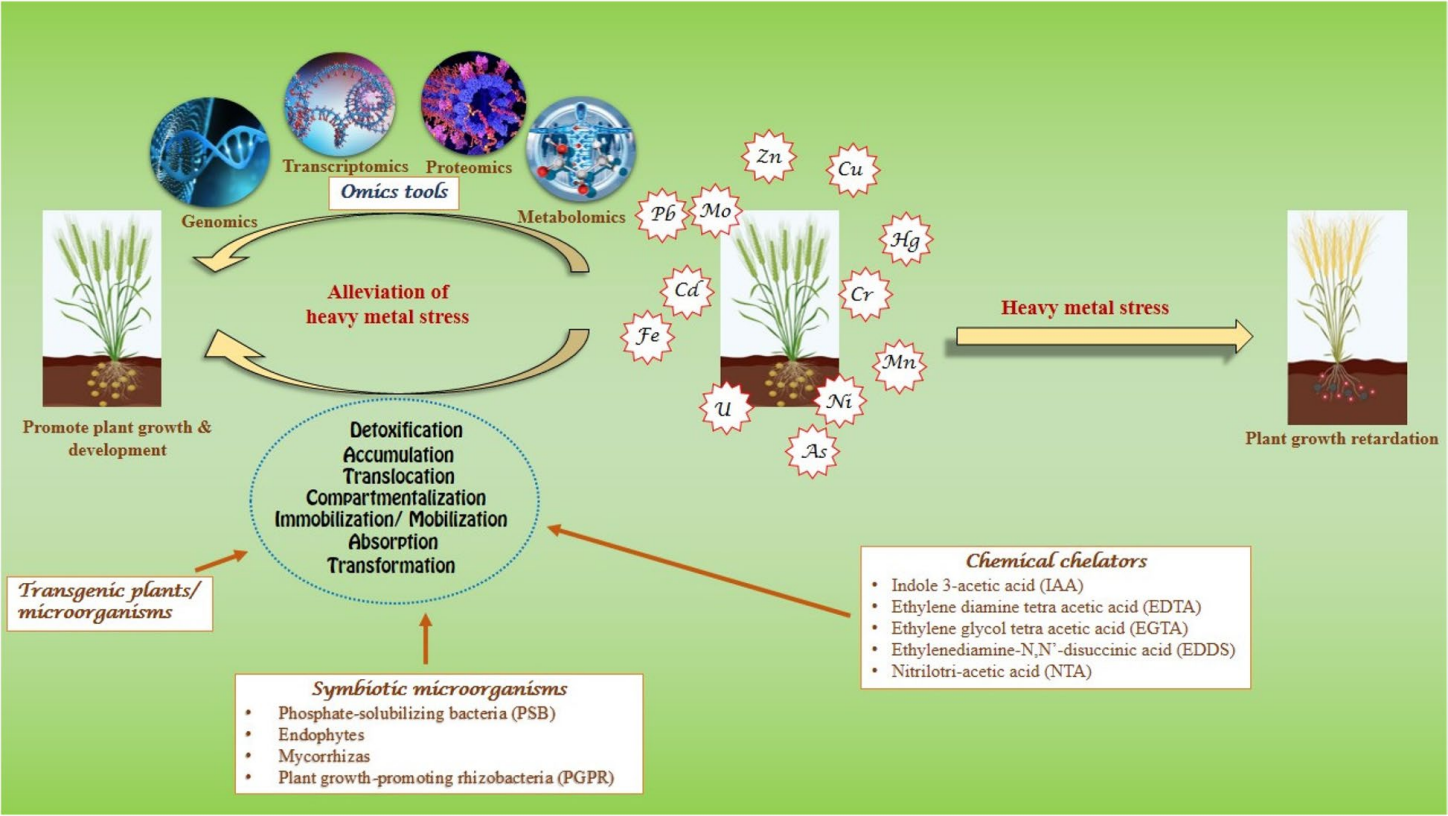
Interaction of different biotic as well as abiotic agents for the amelioration of heavy metal stress in plants

Transcriptome analysis reveals that multiple heavy metals co-regulating unigenes exhibited the function of anti-oxidant enzymes, anti-oxidant substances, transporters, transcription factors and cell wall components (Ge et al. 2022). A comparison of the role of potential genes involved in heavy metal detoxification in *Calotropis gigantea* leaves and root with the aid of comparative transcriptome analysis shows that most of the genes down-regulated in the root but up-regulated in the shoot (Yang et al. 2022a). In heavy metal phytoremediation, metabolite accumulation as part of defensive metabolic pathways performs a significant role. Using metabolomics, metabolic profiling helps to understand the alterations in metabolites and metabolic pathways during heavy metal exposure and their role in heavy metal tolerance (He et al. 2022b; Zou et al. 2022; Anjitha et al. 2023) (Table 6; Fig. 1).

**Table 6:** Various OMICS tool employed in the field of heavy metal phytoremediation potential in plants

Omics tool	Plant	Heavy metal	Result	Reference
Proteomics	*Brassica rapa* L	Cd	Total of 547 succinylated sites on 256 proteins were identified in diverse cellular compartments in the shoots, quantitative analysis show 9 succinylation on 8 protein were altered after 8 h of Cd exposure	Li et al. 2022b
	*Sedum plumbizincicola* X.H. Guo et S.B. Zhou ex L.H. Wu	Cd	Total 3353 membrane proteins identified, total 352 Cd regulated transport proteins identified	Zhu et al. 2022
	*Populus trichocarpa* Hook	Pb	4388 proteins were identified & quantified, among which 36o proteins increased & 182 proteins decreased in abundance upon Pb exposure	Shen et al. 2021
	*Acacia auriculiformis* A. Cunn. ex Benth	Cd	30499 peptides & 6723 identified proteins, of which 5676 were quantified; 59,55 and 83 up-regulated proteins were detected b/w Cd & CK, CdN & CK and CdN & Cd treatments respectively;72,138 & 112 down-regulated proteins were detected b/w Cd & CK, CdN & CK and CdN & Cd treatments respectively (CK-without Cd & N treatment; CdN- Cd & N treatment)	Zhang et al. 2022
	*Brassica campestris* L	Cd	1514 differentially expressed proteins (DEPs) were identified in the Cd treated hairy roots, 451 up regulated proteins and 973 down regulated proteins	Sun et al. 2023
	*Salix matsudana* var. *matsudana f. umbraculifera* Rehd	Cd	655 up-regulated differentially-expressed proteins were identified, proteins that are involved in lignin biosynthesis also up-regulated showing increased lignin content in roots under Cd stress	Yu et al. 2023
Genomics	*Zea mays* L	Cd, Cu & Pb	9 ZmMT genes identified & distributed on 5 chromosomes(1,3,4,6 & 8); most of ZmMTs in root are important for responding to HM stress,but it differ in stem& leaf	Gao et al. 2022
	*Brassica napus* L	Hg, Mn, Cr, Cu, Pb & Zn	33 BnMTPs identified of which 25 BnMTPs unevenly distributed on 13th chromosome & 8 BnMTPs not assigned to specific chromosomes; expression of 24 BnMTPs in leaves & root could respond to HM ion treatment	Xie et al. 2022
	*Populus trichocarpa* Hook	Cd	7 stress related CAX genes identified (PtrCAX 1–7), located on chromosome 1,6,9,11& 16; all CAX genes in root up regulated under Cd stress	He et al. 2022a
		Fe, Mn, Zn & Cd	Total of 11 NRAMP (natural resistance-associated macrophage protein) members (PtNRAMP1–11) were identified, *PtNRAMP* genes were unevenly distributed on six of the 19 *Populus* chromosomes	Ma et al. 2023
	*Eucalyptus grandis* W. Hill ex Maiden	Cu, Cd	20 potential *EgMTPs* (Metal tolerance proteins) genes were identified, *EgMTP* genes were observed to be unevenly distributed on 9 of the 11 chromosomes, upregulation of *EgMTP5*, *EgMTP6*, and *EgMTP11.1* might contribute to the Cu^+2^ and Cd^+2^ transposition via suppression of vacuolar and vesicular ions exposed to excessive metal	Shirazi et al. 2023
Transcripto-mics	*Sedum alfredii* Hance	Cd, Zn, Pb & Cu	295630 unigenes & 597113 transcripts were identified; 20280, 874, 2168 & 6979 unigenes differentially expressed in roots under Cd, Zn, Pb & Cu treatments respectively; most unigenes regulated by Cu were enriched in catalytic activity	Ge et al. 2022
	*Calotropis gigantea* L	Cd	176 (31 up-regulated &145 down regulated) and 1618 (479 up-regulated & 1139 down regulated) differentially expressing genes were identified in roots & leaves of Cd treated plant respectively; results indicate oxidative stress initiated in roots whereas in leaves activated several Cd detoxification processes	Yang et al. 2022a, b
	*Brassica campestris* L	Cd	118 differentially expressed genes identified, that are related to Cd tolerance and absorption	Sun et al. 2023
	*Salix matsudana* var. *matsudana f. umbraculifera* Rehd	Cd	Total of 153 up-regulated differentially-expressed genes identified, genes that responsible for lignin biosynthesis up-regulated	Yu et al. 2023
	*Pistia stratiotes* L	Cd	total of 3107 differentially expressed genes identified, 2666 up-regulated genes, and 441 down-regulated genes, which is mainly involved in glutathione metabolism and lignin biosynthesis	Wei et al. 2023
Metabolomi-cs	*Nicotiana* *tabacum* L. (tobacco cultivar Yunyan 87)	Cd	Identify 1013 metabolites in root & 890 metabolites in leaves; exposure to Fe_3_O_4_ or ZnO nanoparticles recovered more metabolites to normal level and reprogrammed critical metabolic pathways under Cd stress	Zou et al. 2022
	*Brassica rapa* L	Cd	509 differentially expressing metabolites(DEM) were identified; more DEM is involved glutathione metabolism pathway in high Cd-stress than in medium Cd stress	Gao et al. 2022
	*Pistia stratiotes* L	Cd	Cd stress affected eight metabolic pathways, involving 27 differentially expressed metabolites, mainly including unsaturated fatty acids, amino acids (phenylalanine), nucleotides, sulfur compounds, and flavonoids	Wei et al. 2023

## Future prospectus

As in view of future prospectus, the upcoming research studies should focus on the advancement to the existing knowledge and data base regarding the tolerant genotypes of plants. Moreover, focus must be also given to tackling the risk regarding the use of certain amendments employed for the enhancement of phytoremediation techniques. Research should also concentrate on how to increase the success rate of transgenic approaches to impart the tolerance towards pollutants. Furthermore, it has been reported that the application of soil amendments helps to improve the soil microflora but it never addressed the query that whether this enhancement in the soil microflora may have any influence on the neighboring plants growing in the soil.

## Conclusion

Phytoremediation is a promising method for decontaminating the polluted environment using plants and the remediation efficiency of plants can be enhanced by the application of symbiotic microorganisms, chemicals and various transgenic approaches. Combined applications of chemicals with other amendments practices increase the efficiency of heavy metal phytoremediation than the independent treatments. But the major limitation is that certain chemicals can negatively affect the plant growth, as it causes phytotoxicity above the optimal level. Even though advancement in modern- sustainable techniques leads to the development of noval approaches for the remediation of heavy metals includes application of the microbial community, invention of transgenic organisms and analysis of omics data. With the aid of modern technologies, we can design potential transgenic organisms to alleviate heavy metal toxicity. Comparison omics tools like genomics, proteomics, metabolomics and transcriptomics with genome editing technique aid to evaluate the functional aspects of genes and proteins involved in heavy metal tolerance. These modern technologies can offer plant and microbes with high remediation potential and high metal stress tolerance for the clearing of polluted land in a short duration.

## Data Availability

Not applicable.

## Abbreviations

ACC deaminase: 1-Aminocyclopropane-1-carboxylate deaminase
PSB: Phosphate-solubilizing bacteria
AMF: Arbuscular mycorrhizal fungi
PGPB: Plant growth-promoting bacteria
PGPR: Plant growth promoting rhizobacteria
IAA: Indole 3-Acetic Acid
EDTA: Ethylene Diamine Tetra Acetic acid
EGTA: Ethylene Glycol Tetra Acetic Acid
EDDS: Ethylenediamine-N, N′-succinic acid
NTA: Nitrilotriacetic acid
MT: Metallothionein
GEOs: Genetically engineered organisms
